# Production Losses From an Endemic Animal Disease: Porcine Reproductive and Respiratory Syndrome (PRRS) in Selected Midwest US Sow Farms

**DOI:** 10.3389/fvets.2018.00102

**Published:** 2018-05-16

**Authors:** Pablo Valdes-Donoso, Julio Alvarez, Lovell S. Jarvis, Robert B. Morrison, Andres M. Perez

**Affiliations:** ^1^Department of Agricultural and Resource Economics, University of California Davis, Davis, CA, United States; ^2^Department of Veterinary Population Medicine, College of Veterinary Medicine, University of Minnesota, Saint Paul, MN, United States; ^3^Center for Animal Health and Food Safety, University of Minnesota, St. Paul, MN, United States

**Keywords:** Porcine Reproductive and Respiratory Syndrome, Production Impacts, Sow Farms, US Swine Industry, Endemic Animal Disease, Fixed effects model

## Abstract

Porcine reproductive and respiratory syndrome (PRRS) is an endemic disease causing important economic losses to the US swine industry. The complex epidemiology of the disease, along with the diverse clinical outputs observed in different types of infected farms, have hampered efforts to quantify PRRS’ impact on production over time. We measured the impact of PRRS on the production of weaned pigs using a log-linear fixed effects model to evaluate longitudinal data collected from 16 sow farms belonging to a specific firm. We measured seven additional indicators of farm performance to gain insight into disease dynamics. We used pre-outbreak longitudinal data to establish a baseline that was then used to estimate the decrease in production. A significant rise of abortions in the week before the outbreak was reported was the strongest signal of PRRSV activity. In addition, production declined slightly one week before the outbreak and then fell markedly until weeks 5 and 6 post-outbreak. Recovery was not monotonic, cycling gently around a rising trend. At the end of the study period (35 weeks post-outbreak), neither the production of weaned pigs nor any of the performance indicators had fully recovered to baseline levels. This result suggests PRSS outbreaks may last longer than has been found in most other studies. We assessed PRRS’ effect on farm efficiency as measured by changes in sow production of weaned pigs per year. We translated production losses into revenue losses assuming an average market price of $45.2/weaned pig. We estimate that the average PRSS outbreak reduced production by approximately 7.4%, relative to annual output in the absence of an outbreak. PRRS reduced production by 1.92 weaned pigs per sow when adjusted to an annual basis. This decrease is substantially larger than the 1.44 decrease of weaned pigs per sow/year reported elsewhere.

## 1. Introduction

The effects of natural events such as a disease outbreak are typically difficult to measure since simultaneous shifts can occur along several dimensions. The analysis of longitudinal data may reveal dynamic change that would be hard to recognize when only cross-sectional data are used (1, 2). With panel data, one can examine when depression on production occurs -if there is any-, either at the time of the outbreak, soon after or even before, and for how long such depression occurs. Previous studies have addressed the impact of porcine reproductive and respiratory syndrome (PRRS), but have not provided detailed information regarding how the disease affected farm performance. Here, we used longitudinal data routinely collected from sow farms from a US firm between 2014 and 2015 to explore the intensity and extension of outbreaks of PRRS. We then evaluated the effects on revenue due to a decrease on output production using the pre-, during, and post-outbreak periods. This approach allows us to evaluate if outbreaks were reported on time, as well as the extension and length of the impact on production.

Endemic animal diseases can affect farm profit by reducing output, increasing production costs, and reducing product price (3, 4). For example, PRRS, which was first identified in the 1980s, has become one of the most important endemic animal diseases in the US (5–10). It affects the swine industry and disease control is difficult due to factors inherent to the disease and the nature of the swine production system. The causal agent is an RNA-virus (PRRSV) from the *Arteriviridae* family that is highly mutagenic and resistant to the low temperatures registered in Midwestern areas of the US, where a significant proportion of the US swine industry is located. On the other hand, the disease is highly transmissible and can persist for long periods in chronically infected animals and in the environment, if contaminated through secretions and excretes (10–12). Because PRSS has no effect on humans and has little impact on international trade, PRRS is a non-reportable disease in the US. There are no official programs for its control, but producers in some regions have begun collaborative programs to exchange information on PRRS outbreaks in the hope that coordinated action might reduce disease effects (13).

PRRSV spreads between and within farms via airborne transmission, the introduction of infected animals and contaminated fomites, often associated with the failure of biosecurity protocols (5, 10, 14–16). PRRS may increase abortion and mortality rates in pre- and post-weaning pigs, lead to reproductive failure in sows, and lower feed conversion in feeder pigs (17–19), thus affecting several stages of the swine production cycle. However, the severity and length of the impact at each production stage are still unclear.

In high farm density areas, PRRSV eradication is not the main target. Indeed, farmers prefer to maintain homogeneous levels of immunity in breeding herds using vaccination or, although less common, exposing animals to live virus (10). Herd closure and rollover is one of the most common strategies to eradicate PRRSV from sow farms. It consists in stopping introducing new sows as replacements in addition to remove seropositive animals for at least 24 weeks (20). A study showed that production of PRRSV-negative pigs was reached 27 weeks after herd closure started, although an important variation between farms was observed (21). Alternatively, whole-herd depopulation and repopulation strategy is the most effective strategy described but in many cases is financially impracticable (10).

Two studies have estimated the economic impact of PRRS using data from a set of farms and then extrapolating their results to the entire US swine industry (7, 9). They reported total annual losses of ~$560 million and ~$664 million, respectively (7, 9). Although the two studies estimated losses similar in magnitude, they significantly differed in the proportion attributed to losses on sow farms. While Holtkamp et al. estimated that 46% of total losses ($302 million) occurred on sow farms (9), Neumann et al. estimated that only 12% of total losses ($67 million) occurred on sow farms. The causes of the differences in their loss estimates are not explicitly explained, but may occur because of differences in the epidemiology of the disease at different times, the diversity of clinical outputs in infected animals and/or differences in types of farms.

We observe that the effects of disease vary slightly across the farms in our study despite a common management approach, but the availability of data from multiple farms is likely to provide a better estimate of impact than would the use of data from only one farm. We measure the effect of disease using data prior to the outbreak as the baseline and find that disease impact varies over time, with output declining rapidly initially following the outbreak and then recovering slowly and non-monotonically. We gain additional insights into the progression and recovery of disease by measuring changes in seven other performance indicators. Our methodology can be used to characterize disease impact at the farm and/or firm level, as it provides information on the timing of disease effects, the pathways through which PRRS affects production, and the total time needed for recovery. We anticipate that the results presented will help in the development of more accurate models for evaluating alternative PRRS prevention and control strategies in the US.

## 2. Materials and Methods

### 2.1. Study Population

We screened production records from a large, vertically integrated swine firm that includes farms in each stage of the swine production cycle, i.e., breeding and growing. All of the farms are located in the Midwestern region of the US. Numerous of the sow farms (i.e., weaned pigs suppliers within the firm) experienced PRRS outbreaks during 2014–2015. An outbreak was reported when animals showed PRRS-compatible clinical signs that were subsequently confirmed through PCR testing. We chose for analysis only sow farms that had not experienced a PRRS outbreak for at least one year prior to the outbreak studied in this analysis. In addition, we excluded from the analysis any farm that experienced cases of porcine epidemic diarrhea virus during the eight months before the PRRS outbreak to avoid confounding disease effects. All sow farms in this firm routinely applied the same commercially available modified live vaccine (MLV). Thus, all farms were classified as positive-stable without undergoing elimination (i.e., Category 2A according to the American Association of Swine Veterinarians (22) at the time of this study. This firm also used a common disease management protocol for all its farms.

We measured disease impact on output using the weekly number of weaned pigs (WP), which subsequently was used to estimate the decline in the value of output due to a PRRS outbreak. Likewise, we used weekly data for seven statistics, referred here as performance indicators, to more comprehensively assess how the disease affected weaned pig output. These indicators are: the number of live births per litter -or litter size- (LS), the number of stillbirths per litter (SB), the number of pre-weaned pigs dead (PWM), the number of sows dead (SM), the number of sows aborting (AB), the number of sows with repetition of service (RE), and the number of sows farrowing (FA).

### 2.2. Data Analysis

From each farm, we obtained longitudinal data for 48 weeks for the number of weaned pigs, the count data for the seven performance indicators, and the number of sows in the farm. Weekly data include 12 weeks prior to the reported outbreak, the week in which the outbreak was reported, and 35 subsequent weeks. For each farm, the week in which the PRRS outbreak is reported is labeled as week *t*, while the 12 weeks before were labeled as *t *− 12, *t *− 11… *t *− 1, respectively, and the 35 weeks after the outbreak were labeled as *t* + 1…*t* + 35.

For each *i^th^* farm (*i* = 1, 2, 3… 16), we took the logs of eight dependent variables (weekly counts of weaned pigs and the seven performance indicators) to evaluate different effects of the outbreak. Adjusting by seasons and the number of sows in each farm, we used a log-linear fixed effects approach to estimate (1) a baseline for the production of weaned pigs during the pre-output period, i.e., between *t* − 12 and *t *− 1; and (2) the weekly proportional change in production within each farm. The estimated baseline is used to measure PRRS’ effect on production after the outbreak, i.e., between *t* and *t* + 35. We then used the same procedure to analyze, separately, the baseline values and post-outbreak effects for each performance indicator (See details in sections 2.3 and 2.4).

Using longitudinal data allows us to reveal PRRS dynamics that might be difficult to identify if using cross-sectional data. In this case we evaluate the net effect of the outbreak on production within a selected set of farms. The use of fixed effects also permits us to manage the unobserved heterogeneity within farms (e.g., internal management, location, prevalence of chronic disease, and types of buildings, ventilation and acclimation systems) whose omission could bias the estimated coefficients. We assume that time-invariant effects are unique to each farm and are not correlated with effects on other farms. In addition, the expectation that individual farms have stable characteristics over time and the recognition our sample set has not been selected randomly led us to prefer a fixed effects rather than a random effects model. We used the Hausman test to determine whether the unobserved effects are distributed dependently of the regressors (1, 2, 23). We used Stata Statistical Software V13.1 to perform all these statistical computations and graphic designs (24).

### 2.3. Estimation of Production and Performance Baselines

Selected sow farms routinely supply weaned pigs to growing farms that belong to the same vertically integrated firm. Thus, we hypothesized that the weekly supply of piglets from each farm was stable in the absence of externals shocks, such as a disease outbreak, i.e., that piglet production during the pre-outbreak period would show no significant time trend. We test this hypothesis using equation (1):

(1) $$Y_{itk}=\mu_{0}+\sum_{j=1}^{2}\beta_{j}X_{ijt}+\delta t+\alpha_{i}+\varepsilon_{it}$$

*Y_itk_* is the *k^th^* dependent variable (*k* = WP, PWM, LS, SB, RE, FA, AB, SM) that has been log-transformed so that the estimated coefficient indicates the proportional change in output per unit change in any independent variable. As noted, equation (1) was used to estimate equations for production and each of the seven performance indicators as well. *X_ijt_* is a vector that controls for observable independent variables that may vary across time; we used seasons (summer, fall, winter and spring) and the number of sows (as a proxy for farm size). The term *t* denotes a trend whose estimated coefficient (*δ*) should be close to zero if output is stable. Finally, *α_i_* denotes unobservable differences across farms, and *ε_it_* a week random disturbance term that is independent of the explanatory variables, and *α_i_* and μ_0_ yield the intercept for each farm.

### 2.4 Estimation of PRRS Impacts

To estimate the impact of PRRS, we used equation (2), which is similar to equation (1) except that the trend term (*t*) was replaced by a set of dummy variables (*T_t_*), one for each time period, introducing a time fixed effect. *T_t_* was set to 0 for the first observation (*t* − 12). The estimated *δ* coefficients provide a direct estimate of the weekly changes for each week subsequent to week* t* − 12 in production and in each of the performance indicators, and also providing measures of the severity and the duration of the outbreak’s effects. Again, the coefficients on the log-transformed dependent variables indicate the proportional change in the dependent variable associated with a unit change in the independent variable.

(2) $$Y_{itk}=\mu_{0}+\sum_{j=1}^{2}\beta_{j}X_{ijt}+\sum_{t=2}^{48}\delta_{t}T_{t}+\alpha_{i}+\varepsilon_{it}$$

**Figure 1:**
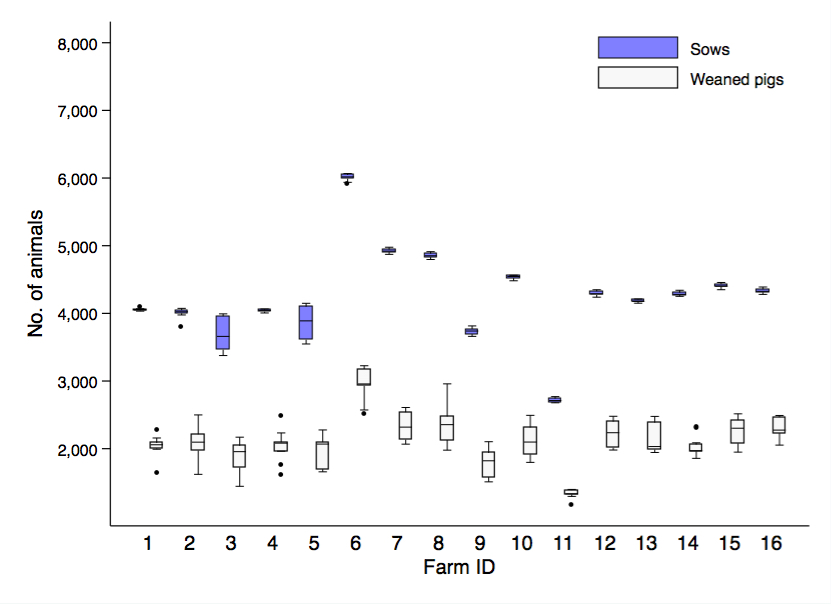
Number of sows and weaned pigs in sixteen farms affected by porcine reproductive and respiratory syndrome (PRRS) through the pre-outbreak period. Boxes indicate the first and third percentile and middle bars represent the median.

As in equation 1, *X_ijt_* represents the vector of observable time-variant covariates, such as seasons and the number of sows on each farm. We used fitted values from equation 2 to estimate the profile of disease impact on weaned pig production, from initial impact through recovery. The estimated coefficients from equation 2 can be used to predict output for each of the 16 farms for each of the pre- and post-outbreak weeks. To obtain a graphical representation of the post-outbreak’s profile, we fit a 4th degree polynomial function to the mean estimates of weekly farm output. Figure 1 shows the mean estimate of aggregated weaned pig production on the 16 farms.

We used a Wald test to verify that of the dummy coefficients for the time fixed effects for all weeks (*T_t_*) are equal (i.e., H_0_) or different than 0 (i.e., H_1_). If the estimated weekly coefficients are jointly equal to zero (*P* > 0.05), there is no need to include *T_t_* into the model.

We followed the same procedure, separately, for each performance indicator. We anticipated that the duration of the outbreak would end when each dependent variable approached a value similar to its baseline in the pre-outbreak period.

### 2.5 Production Efficiency

We measured the change in sow efficiency, ΔE, caused by a PRRS outbreak, by subtracting the number of weaned pigs produced per sow during the year in which we observed PRRS infections from the weaned pigs produced per sow in a theoretical year without an outbreak. We used exponential outputs from equation (1) and (2). The first term on the right hand side of equation (3) corresponds to a theoretical year without an outbreak. During a 52-weeks period (1 year), we divided the predicted baseline ($e^{{\overset{-}{Y}}_{ibk}}$) production of each of the 16 farms [obtained from equation (1)] by the average number of sows on these farms during the pre-outbreak period. The second term on the right side of equation (3) corresponds to a year with an outbreak, which is similar to the first term except that the values for the 36-weeks of the post-outbreak period (including week *t*) correspond to the to the predicted values of production ($e^{{\hat{Y}}_{itk}}$) from equation (2). The output during the 12-week pre-outbreak period and the four-week period after our data set ends is assumed to be equal to the baseline level. To the extent that farm output has not totally  recovered at the end of 35 weeks, this measure slightly underestimates the decline in efficiency due to a PRRS outbreak.

(3) $$\Delta E_{ik}=\frac{52e^{{\overset{-}{Y}}_{ibk}}}{{\overset{-}{n}}_{i}^{sow}}-\left(\frac{16e^{{\overset{-}{Y}}_{ibk}}+\sum_{t=0}^{35}e^{{\hat{Y}}_{itk}}}{{\overset{-}{n}}_{i}^{sow}}\right)$$

The subscript *b* denotes the extrapolation of baseline period values obtained from equation (1); while *t* represents estimated values during the post-outbreak period for each farm *i* obtained from equation (2). As Equation 3 yields a measure of the change in weaned pigs produced per sow, we subsequently converted this estimate into an estimated percentage decline in efficiency, i.e., %ΔE, dividing the second term in equation 3) by the first term minus 1. We then estimated the effects of PRRS on the number of abortions (k = AB), number of repeated services (k = RE), and farrowing (k = FA) per sow using the same approach.

### 2.6 Losses Due to Reduced Weaned Pigs Marketed

We estimated the decrease in farm revenue by multiplying the mean decrease in sow efficiency (weaned pigs per sow) by the mean number of sows per farm and again by the mean market price [$45.2/weaned pig (25)] during the period of analysis. The mean market price was calculated for the period July 2014 to September 2015, the period between the earliest outbreak in the farms studied and the latest month in which we estimated that a PRRS outbreak was affecting weaned pig production. We recognize that farms suffer economic losses from other effects than output reduction, but the lack of data prevented us from evaluating these effects.

## 3. Results

Sixteen farms fulfilled the inclusion criteria for this study, leading to a balanced dataset with 768 weekly observations distributed over 48 successive weeks. All outbreaks occurred during the second half of 2014. During the pre-outbreak period, sow inventory on different farms ranged from 2,714 to 6,009 breeding females (mean = 4,245, SD = 696) (Figure 1). Among our sample, only two farms (ID = 6 and 11) stood out as having unusually large or small sow inventories (Figure 1).

**Figure 2:**
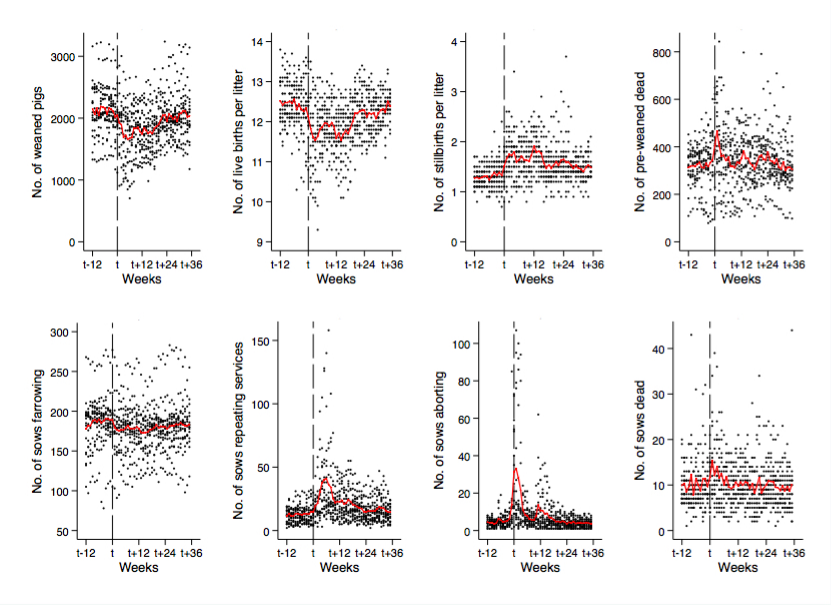
Weekly average (red lines) and observed (dots) number of weaned pigs and the seven additional performance indicators during pre- and post-outbreak period.

Weekly observed values indicated weaned pig production on every farm worsened sharply at or just before the PRRS outbreak, as did the seven performance indicators. That effect lasted for some weeks (Figure 2).

### 3.1. Estimation of Production and Performance Baselines

The estimated *δ*’s were close to zero and/or statistically insignificant (*P* ≥ 0.05) in the regression for pre-outbreak number of weaned pigs (WP) and for nearly all of the performance indicators, indicating pre-outbreak production stability. The exceptions occurred in the regressions for abortions (AB) and litter size (LS) (See Supplementary Material 1,** Table S1**). After analyzing the data, we dropped the observation for *t* *− *1 in the regression for the number of sows aborting and the observations for weeks *t* *− *1 and *t* *− *2 for the number of live births per litter, after which the estimated *δ*’s for those performance indicators were close to zero and insignificant as well. We used the means of the fitted variables for these regressions to establish their respective baselines (Table 1).

**Table 1:** Mean baseline estimates for weaned pigs production and performance indicators

**Statistic**	**WP**	PWM*	**LS**	**SB**	**RE**	**FA**	**AB**	**SM**
**Mean (SE)**	2094.1 (7.055)	304.1 (0.924)	12.5 (0.025)	1.3 (0.007)	11.3 (0.084)	183.5 (0.38)	4.8 (0.104)	9.0 (0.035)

Values indicate means and in parentheses the standard errors (SE) of exponential values obtained from results of 8 separate regressions used to evaluate production and performance [i.e., number of weaned pigs (WP), number of pre-weaned dead (PWM), number of live births per litter (LS), number of stillbirths per litter (SB), number of sows repeating services (RE), number of sows farrowing (FA), number of sows aborting (AB), and number of sow dead (SM)] before the outbreak (equation (1)).

* Farm ID #5 was dropped from PWM due to lack of information.

### 3.2. Estimation of PRRS Impacts

**Figure 3:**
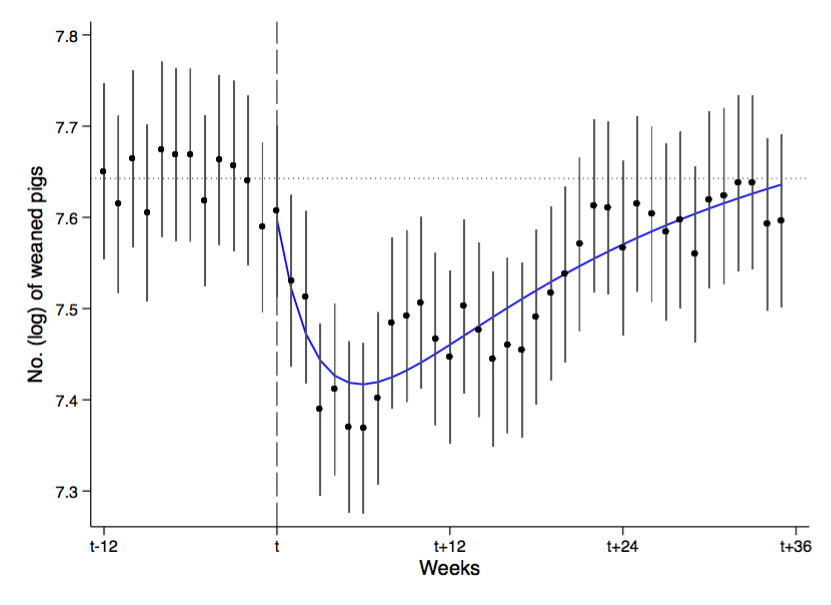
Means of fitted number (log) of weaned pigs during pre- and post-outbreak period (**Table S2)**. The horizontal dotted line shows the estimated overall baseline of production (Table 1). The smooth blue line shows a 4th degree polynomial function that fits estimated means of number (log) of weaned pigs each week during the post-outbreak period (*t* to *t* + 35). Vertical lines indicate 95% confidence intervals.

Dummy coefficients for weeks (*T_t_*) were statistically different than 0 (*P* < 0.05) indicating that the inclusion of *T_t_* as fixed time effects in equation (2) is appropriate. Although we found no significant trend in weaned pig production during the pre-outbreak period, holding the number of sows and season constant when using equation (2), we observed a consistent decrease (14 of 16 farms) in weaned pig production relative to the baseline in the week *t −* 1, immediately before the outbreak was reported in week *t* (See **Table S2** and Figure 3). The decreases ranged between 1 and 12%. As our regressions based on equation 1) showed no significant trend in weaned pig production even when week *t*** *−* **1 was included, we did not remove week *t −* 1 from the baseline period. Had we done so, the baseline would have been very slightly higher and the estimated damages from PRRS slightly greater, as discussed subsequently.

We estimate that aggregate weaned pig production for the 16 farms decreased from the baseline production of 2,094 per week to 1,600 in week *t* + 5, when output was a full 23% lower than the baseline. Table 1. The results show that farm production decreased monotonically from t* −* 1 to *t* + 5, and then began to recover (see Figure 3 and **Table S2**). Output recovered moderately from *t* + 5 until *t* + 11, at which point another significant decline in production occurred to *t* + 17 (see Figure 3 and **Table S2**). Eight of the 16 farms then recovered monotonically to their baseline production levels by *t* + 33, but a slight drop occurred again in *t* + 34 and *t* + 35 with 15 farms producing lower than the baseline. In the aggregate, observed production approached the baseline value by the end of *t* + 35, when our sample ended. Estimated output appears slightly lower than the pre-outbreak level, but the difference is not statistically significant (Figure 3 and **Table S2**).

**Figure 4:**
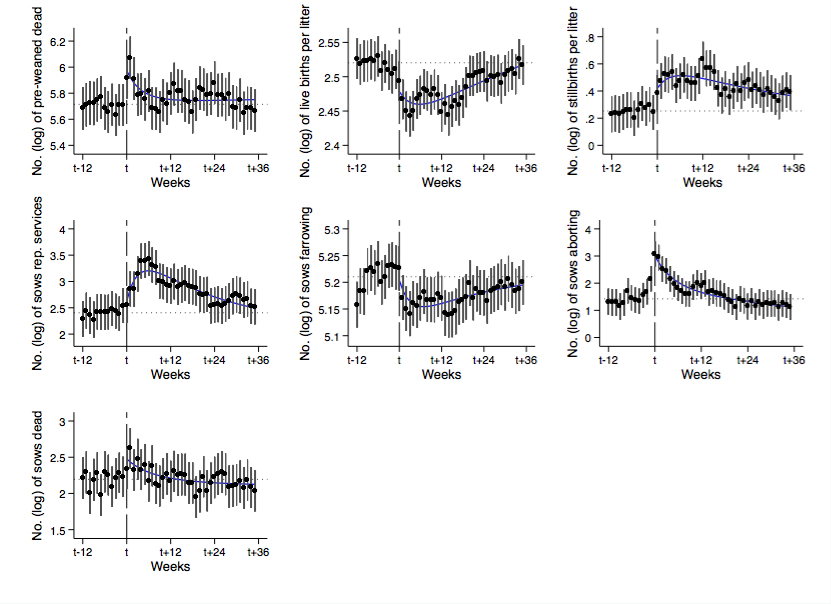
Means of fitted numbers (log) of the seven performance indicators during pre- and post-outbreak period (**Table S2**). The horizontal dotted lines show the estimated baselines of each indicator (Table 1). The smooth blue lines show a 4th degree polynomial function that fits estimated means number (log) for each indicator during the post-outbreak period (*t* to *t* + 35). Vertical lines indicate 95% confidence intervals.

Similar to the production of weaned pigs, the seven performance indicators did not fully recover to their pre-outbreak means. Week-to-week comparisons revealed changes in all performance indicators, with some variation in timing and intensity. For each performance indicator, the recovery of each farm fluctuated around a rising trend estimated for all farms, and again showed a non-monotonic recovery (Figure 4). As expected, some performance indicators presented a lag with respect to the trend observed in weaned pig production. A significant increase in the number of pre-weaned pigs dead was detected at *t* with an average expected rise of 79 (SE = 19) deceased animals relative to the baseline, reaching a maximum increase at *t* + 1, with 143 expected extra losses (SE = 23) (Figure 4). While litter size did not show a significant decline at t, the expected number of live births decreased by around 1 animal between *t* + 1 and *t* + 18, reaching a maximum decline at *t* + 2 and *t* + 3 and a new deterioration at *t* + 14. The number of stillbirths increased between *t* and *t* + 16, reaching a maximum at *t* + 12, with 2 stillbirths per litter (see **Table S2** and Figure 4). Although there was no immediate increase in the number of sows designated for repeated service the week of the outbreak report, by *t* + 6 the number of sows that were designated to repeat service increased from 11 sows in the estimated baseline to 31 sows. Likewise, the number of pigs farrowed declined after *t* + 1. The number of abortions significantly increased at week *t* *− *1, doubling the number of sows that aborted prior to *t −* 1. The number of sows with abortions peaked in the week of the outbreak report (i.e., at *t*) at a level five times higher than the baseline level. Finally, sow mortality showed a significant increase in *t* + 1 with one more sow death than during the baseline period (see **Table S2** and Figure 4). In general, the indicators confirm that a PRRS outbreak affected several production stages (e.g., pre-mated sow, pregnant sow, and piglets) for an extended period of time (see **Table S2**).

### 3.3. Production Efficiency

Our estimates indicate that a PRRS outbreak caused a 7.4% (min = 4.1%, max = 13.4%) decrease in weaned pigs per sow year, i.e., 1.92 (min = 1.05, max = 3.18) fewer weaned pigs per breeding unit. The causes of the decrease can be seen in the performance indicators. There was a slight decrease (2.4%) in the number of sows farrowing per year, from 2.3 to 2.2 farrows per sow year. In an average sized farm of this firm (i.e., 4,245 sows, see Figure 1), the slight reduction in farrowing yielded a decline of 249 fewer farrows per year. The chances that a sow repeats service increased by 37%, while aborted fetuses increased by 26% in a year with a PRRS outbreak (Table 2).

**Table 2:** Estimated means of sow efficiency comparing years in absence and presence of a PRRS outbreak.

**Indicator**	Year without PRRS*	**Year with PRRS^*†*^**	**%ΔE**
**No. of weaned pigs produced per sow year**	25.95 (0.539)	24.03 (0.515)	−7.4%
**No. of farrowing per sow year**	2.29 (0.072)	2.23 (0.034)	−2.4%
**No. of repeated service per sow year**	0.15 (0.015)	0.20 (0.005)	36.9%
**No. of abortions per sow year**	0.05 (0.006)	0.07 (0.002)	25.7%

*Values indicate means and in parentheses the standard errors obtained from the first term of the right side of equation (3).

† Values indicate means and in parentheses the standard errors obtained from the second term of the right side of equation (3).

### 3.4. Timing of Outbreak

The decline in weaned pigs marketed in week *t* − 1, although statistically insignificant, as well as changes in some performance indicators (e.g., number of sows aborting), suggest that the outbreak may have started in week *t* − 1, one week before it was reported. We therefore developed an alternative estimate of production losses that can be compared to the estimated loss if the outbreak is assumed to begin in week *t*. Eliminating *t* − 1 from the pre-outbreak period led to estimation of a slightly higher baseline and, as a result, to a higher estimate of PRRS losses. Nonetheless, the difference between this estimate and our primary estimate is very small. Our primary estimate (using 12 weeks as pre-outbreak period) is that PRRS reduced weaned pig production per farm by 7.4% on an annual basis, leading to a decrease in output value per sow year of $86.6, or $367,521 per farm year for an average sized farm. If instead we assume the outbreak began in *t* −1 (i.e., using 11 weeks as pre-outbreak period), the estimated reduction in weaned pig production was 7.6%, or $88.8 less per sow year and an average revenue loss of $376,773 among the farms studied.

## 4. Discussion

We analyzed the impact of a PRRS outbreak on weaned pig production in a set of sow farms that are part of the same swine firm in the US. We estimated the time profile of disease effects, identifying the weekly changes in output relative to a pre-outbreak baseline. We find that PRRS caused a 7.4% decline in production value measured over a one-year period. Correspondingly, PRRS reduced production by 1.92 weaned pigs per sow when adjusted to an annual basis. This decrease is substantially larger than the 1.44 decrease of weaned pigs per sow/year reported in another study (9). We note that total losses due to PRRS are likely to be greater than the revenue losses estimated in this study, as total losses must include cost increases associated with the disease, e.g., an increase in management expenses, biosecurity investments, additional feed and veterinary inputs, plus a possible decrease in the weight or in the sales price of piglets (4).

We found that weaned pig production declined in week *t *− 1, although statistically insignificant, as did several performance indicators. The data suggest that the average PRRS outbreak in this set of farms began at least one week before it was announced. This delay may be explained, at least in part, by the inability of producers to detect PRRS until animals begin to show explicit clinical signs, as well as the additional time needed to test and confirm the disease. The lag between the outbreak of disease and the appearance of clinical signs may be longer in farms using vaccination programs, as in our sample, where clinical signs may be subtle (26–31). It seems likely that some weaned pigs being shipped by these farms in week *t* − 1, when the disease was almost certainly present in these farms, but as yet unannounced, were infected with PRRS. The relatively slow identification of the disease means that animal movements out of infected premises must be a common source of disease spread. This is particularly important in sow farms that deliver wean pigs to different swine grower facilities each week. Reducing disease spread via movements of diseased animals might significantly reduce overall losses to PRRS (10).

The rise in abortions was the strongest signal of PRRSV activity in our data. Increased surveillance, particularly to rising abortions, may allow farms to identify PRRS more quickly. Abortions were rising in the several weeks prior to the reporting of the outbreak in some of the farms in the sample. Abortions rose significantly in *t* − 1 and then increased sharply in week *t*. The number of abortions declined rapidly and fairly monotonically following week *t*, with a slight uptick in weeks *t* + 10 to *t* + 13, and recovered to the baseline level by about week *t* + 20. Thus, to the extent that abortions are an indicator of an active virus in the sow herd, circulation of the virus appears to have ended about 20 weeks after it was reported. The uptick in weeks *t* + 10 to *t* + 13 suggests that the disease may have been infecting other susceptible cohorts of sows within the farms two to three months after the initial outbreak.

The length of PRRS outbreaks, as well as their effects over time, is highly variable. For example, one study estimated effects of an outbreak during 12 weeks post detection (32), while another indicated that production of negative piglets was reached 27 weeks post infection (21). Our results demonstrate that PRRS has a negative effect on weaned pig production for a longer time than previously estimated. In our study, the estimated means of weaned pig production remained below the baseline throughout the 35 weeks that we are able to observe following the outbreak. Although the production of weaned pigs recovered to a level that is not significantly different (*P* < 0.01) from the baseline, we cannot definitively declare that there was no effect beyond week *t* + 35. Nonetheless, it appears that any continued effect is likely to be very small relative to the large effect occurring before week *t* + 35.

We detected a consistent decrease in production until the 5th week after the outbreak report, followed by a non-monotonic recovery. All performance parameters followed a similar non-monotonic recovery pattern. Each indicator manifested a sharp worsening after the outbreak, followed by partial recovery and at least one mild period of deterioration. The dynamic up-and-down impact of PRRS on weaned pig production was surprising. The precise causes are unclear, but the disease may spread more slowly and unevenly through the sow herd than anticipated, particularly on large units with multiple cohorts, in addition to possible incoming flows of replacement sows. This effect might also explain the longer period of recovery in our study, versus another study that found production returned to the baseline in 16.5 weeks for cohorts vaccinated with an MLV and using herd closure as a control strategy (21).

Other performance indicators provided consistent signals. Pre-weaning mortality increased sharply in weeks *t* − 1 to *t* + 1, declined to pre-outbreak levels by *t* + 10, and then oscillated about that level until about *t* + 24. Sow mortality increased in week *t* + 1 and remained above baseline levels until week *t* + 5. The increase in sow mortality could affect the age structure of the herd and consequently its production. Stillbirths increased until week *t* + 12, indicating that some infected sows carried damaged fetuses to birth. The number of stillbirths remained elevated through *t* + 36, suggesting that infected sows may have a higher probability of producing stillborn piglets for more than one pregnancy. The failure to conceive was followed by repeated services, which must have contributed to the lag in weaned pig production in later weeks. The numbers of pigs aborting or dying indicated that PRRS had its strongest effects on fetuses. PRRS kills relatively few sows and piglets, though the economic damage from sow mortality and/or their subsequently reduced productivity is important.

Information regarding the strains of PRRS virus that affected each farm was not available for this study, as systematic sequencing of PRRS virus following outbreaks is still scarce. More than one strain might affect a given area, although in general genetic variation is more related to temporal rather than spatial variation (10). Using a sample of 16 farms may help capture the variability of PRRS outbreaks in the industry, assuming different strains may be affecting different farms.

According to a number of studies, no vaccine prevents PRRS infection, but vaccination may reduce the risk of infection and may also reduce the intensity of outbreaks by reducing the amount of virus excreted by ill animals (26–31). Therefore, our results may show smaller damages than those that would be obtained for farms that do not vaccinate. Similarly, because the farms analyzed in this study belong to a firm with standardized protocols for disease management, our measure of PRRS’ impact could be smaller than would be measured on farms with poorer protocols.

We developed and used a straightforward approach to quantify the dynamic effect of PRRS on weaned pig production within sow farms. We found that PRRS decreased weaned pig production for at least 35 weeks among the firms studied. The magnitude of PRRS’ impact, as expressed in the duration and magnitude of the output decline, were both greater than anticipated. We found that recovery oscillated about a rising trend, i.e., recovery does not depict a clear monotonic increase in production, suggesting that farms suffered from a continuing circulation of the disease within the herd and/or a lingering effect on sows and piglets. Analysis of the underlying performance indicators provided additional insight regarding how PRRS affects farm output over time. Previous studies have utilized numerous assumptions to develop estimates of the total annualized losses to the swine industry due to PRRS (7, 9). We have not attempted to replicate those studies. However, our results suggest PRRS may cause significantly higher losses on sow farms than has been estimated previously. Further, we believe that the losses identified in our farm sample are likely to be smaller than those on the average sow farm infected with PRRS. Nonetheless, we found substantial variation in performance among even a set of relatively standardized 16 farms. There is thus need for caution when using simple averages, as we often have done, rather than distributions across farms.

## Author Contributions

All the authors have met the four criteria described in the guidelines: PV-D designed analyses and data interpretation, revised and approved the version to be published, and agreed to be accountable for all aspects of the work. LJ, JA, RM, and AP revised and approved the version to be published, and agreed to be accountable for all aspects of the work.

## Conflict of Interest Statement

The authors declare that the research was conducted in the absence of any commercial or financial relationships that could be construed as a potential conflict of interest.
