# Supplementary material for: Production Losses From an Endemic Animal Disease: Porcine Reproductive and Respiratory Syndrome (PRRS) in Selected Midwest US Sow Farms
Source: Front Vet Sci. 2018 May 16;5:102. doi: 10.3389/fvets.2018.00102 (PMC5996871; doi:10.3389/fvets.2018.00102)
Supplement: Supplementary file 1 [file Table_1.DOCX]

Supplementary Material

**Production losses from an endemic animal disease: Porcine reproductive and respiratory syndrome (PRRS) in selected Midwest US sow farms**

**Pablo Valdes-Donoso^*^, Julio Alvarez, Lovell Jarvis, Robert Morrison, Andres Perez**

***Correspondence:** Pablo Valdes-Donoso**:** pvaldesdonoso@ucdavis.edu

# Supplementary Tables

Table 1: Coefficients of log-linear fixed effects models for the number of weaned pigs produced and seven performance indicators during pre-outbreak period

| **Variable** | **WP** | **PWM^1^** | **LS** | **SB** | **RE** | **FA** | **AB** | **SM** |
| --- | --- | --- | --- | --- | --- | --- | --- | --- |
| **Time (*t*)** | -0.001 (0.003) | -0.012 (0.006) | -0.004*** (0.001) | -0.004 (0.004) | 0.024 (0.013) | 0.002 (0.002) | 0.038* (0.018) | 0.004 (0.012) |
| **Spring^2^** | 0 | 0 | 0 | 0 | 0 | 0 | 0 | 0 |
| **Summer** | 0.016 (0.047) | 0.173 (0.114) | 0.019 (0.015) | 0.079 (0.074) | 0.096 (0.217) | 0.145*** (0.028) | -0.472 (0.3) | -0.11 (0.205) |
| **Fall** | -0.025 (0.056) | 0.213 (0.136) | 0.03 (0.018) | 0.122 (0.089) | -0.129 (0.260) | 0.126*** (0.0330) | -0.21 (0.360) | -0.012 (0.246) |
| **Winter** | -0.071 (0.089) | 0.586** (0.209) | 0.011 (0.029) | 0.425** (0.141) | -0.108 (0.412) | 0.118* (0.053) | 1.182* (0.57) | 0.095 (0.39) |
| **No. of sows** | 0.0001  (0.0001) | 0.0001 (0.0003) | 0.0001** (0.000) | 0.0002 (0.0002) | -0.0003 (0.001) | 0.0001 (0.0001) | 0.001 (0.001) | -0.0001 (0.001) |
| **Intercept** | 7.136*** (0.48) | 5.124*** (1.28) | 1.978*** (0.156) | -0.734 (0.759) | 3.719 (2.227) | 4.787*** (0.286) | -1.094 (3.081) | 2.777 (2.106) |
| **Within groups error** | 0.082 | 0.309 | 0.086 | 0.168 | 0.478 | 0.141 | 0.431 | 0.388 |
| **Overall error** | 0.095 | 0.217 | 0.031 | 0.151 | 0.442 | 0.057 | 0.612 | 0.418 |
| **No. Obs.** | 192 | 180 | 192 | 192 | 192 | 192 | 192 | 192 |
| **No. Groups** | 16 | 15 | 16 | 16 | 16 | 16 | 16 | 16 |

Table considers 8 separate log-linear fixed effects regressions (equation (1)) for the number of weaned pigs (WP), number of pre-weaned dead (PWM), number of live births per litter (LS), number of stillbirths per litter (SB), number of sows repeating services (RE), number of sows farrowing (FA), number of sows aborting (AB), and number of sow dead (SM). Numbers indicate estimates; standard errors are reported between parentheses. Asterisk (*), double asterisk (**), and triple asterisk (***) denote 5%, 1%, and 0.1% of significance levels respectively. ^1^ Farm ID #5 was dropped from PWM due to lack of information. ^2^ Spring is used as reference category of season.

Supplementary Table 2: Coefficients of log-linear fixed effects models for the number of weaned pigs produced and seven performance indicators

| **Variable** | **WP** | **PWM^1^** | **LS** | **SB** | **RE** | **FA** | **AB** | **SM** |
| --- | --- | --- | --- | --- | --- | --- | --- | --- |
| ***t-12*^2^** | **0** | **0** | **0** | **0** | **0** | **0** | **0** | **0** |
| ***t-11*** | -0.04 (0.05) | 0.02 (0.085) | -0.01 (0.015) | 0.01 (0.068) | 0.15 (0.176) | 0.02 (0.022) | -0.03 (0.247) | 0.07 (0.149) |
| ***t-10*** | 0.01 (0.05) | 0.03 (0.085) | 0.00 (0.015) | 0.00 (0.068) | 0.06 (0.176) | 0.01 (0.022) | -0.03 (0.247) | -0.21 (0.149) |
| ***t-9*** | -0.04 (0.05) | 0.02 (0.085) | 0.000 (0.015) | 0.02 (0.068) | -0.04 (0.176) | 0.05* (0.022) | -0.25 (0.247) | -0.05 (0.149) |
| ***t-8*** | 0.03 (0.05) | 0.05 (0.085) | 0.000 (0.015) | 0.03 (0.068) | 0.1 (0.176) | 0.05* (0.022) | -0.11 (0.247) | 0.04 (0.149) |
| ***t-7*** | 0.02 (0.05) | 0.06 (0.085) | -0.01 (0.015) | 0.03 (0.068) | 0.08 (0.176) | 0.04 (0.022) | 0.31 (0.247) | -0.27 (0.149) |
| ***t-6*** | 0.02 (0.05) | -0.03 (0.085) | 0.00 (0.015) | -0.03 (0.069) | 0.05 (0.177) | 0.06* (0.022) | -0.02 (0.248) | 0.03 (0.15) |
| ***t-5*** | -0.02 (0.05) | -0.05 (0.085) | -0.02 (0.015) | 0.04 (0.069) | 0.04 (0.177) | 0.02 (0.022) | -0.15 (0.248) | -0.03 (0.15) |
| ***t-4*** | 0.02 (0.05) | 0.00 (0.085) | -0.01 (0.015) | 0.08 (0.069) | 0.1 (0.178) | 0.02 (0.022) | -0.19 (0.249) | -0.2 (0.15) |
| ***t-3*** | 0.01 (0.051) | -0.09 (0.086) | -0.02 (0.015) | 0.05 (0.069) | 0.03 (0.178) | 0.04 (0.022) | -0.02 (0.25) | -0.1 (0.151) |
| ***t-2*** | 0.000 (0.051) | -0.02 (0.086) | -0.02 (0.015) | 0.07 (0.069) | -0.03 (0.178) | 0.04 (0.022) | 0.13 (0.25) | -0.02 (0.151) |
| ***t-1*** | -0.04 (0.051) | -0.01 (0.086) | -0.01 (0.015) | 0.01 (0.069) | 0.12 (0.178) | 0.04 (0.022) | 0.63* (0.25) | -0.07 (0.151) |
| ***t*** | -0.02 (0.051) | 0.19* (0.086) | -0.03* (0.015) | 0.15* (0.069) | 0.10 (0.179) | 0.04* (0.022) | 1.47*** (0.251) | 0.02 (0.151) |
| ***t+1*** | -0.08 (0.051) | 0.34*** (0.086) | -0.05** (0.016) | 0.22** (0.07) | 0.4* (0.18) | -0.01 (0.023) | 1.38*** (0.252) | 0.30* (0.152) |
| ***t+2*** | -0.1 (0.051) | 0.18* (0.086) | -0.07*** (0.016) | 0.27*** (0.07) | 0.39* (0.18) | -0.03 (0.023) | 0.95*** (0.252) | 0.00 (0.152) |
| ***t+3*** | -0.22*** (0.051) | 0.05 (0.087) | -0.07*** (0.016) | 0.26*** (0.07) | 0.68*** (0.18) | -0.04 (0.023) | 0.88** (0.252) | 0.15 (0.152) |
| ***t+4*** | -0.19*** (0.051) | 0.06 (0.087) | -0.06*** (0.016) | 0.27*** (0.07) | 0.91*** (0.181) | -0.02 (0.023) | 0.63* (0.254) | 0.01 (0.153) |
| ***t+5*** | -0.23*** (0.051) | 0.02 (0.087) | -0.05** (0.016) | 0.17* (0.07) | 0.92*** (0.181) | -0.03 (0.023) | 0.43 (0.254) | 0.08 (0.153) |
| ***t+6*** | -0.22*** (0.052) | 0.08 (0.087) | -0.04* (0.016) | 0.21** (0.071) | 0.93*** (0.182) | -0.01 (0.023) | 0.22 (0.255) | -0.18 (0.154) |
| ***t+7*** | -0.18** (0.052) | -0.06 (0.088) | -0.03 (0.016) | 0.24** (0.071) | 0.8*** (0.183) | 0.01 (0.023) | 0.13 (0.256) | 0.04 (0.155) |
| ***t+8*** | -0.09 (0.052) | -0.06 (0.088) | -0.03 (0.016) | 0.19** (0.071) | 0.74*** (0.183) | 0.00 (0.023) | 0.02 (0.257) | -0.21 (0.155) |
| ***t+9*** | -0.08 (0.052) | -0.08 (0.088) | -0.03 (0.016) | 0.17* (0.071) | 0.48** (0.183) | 0.01 (0.023) | 0.01 (0.256) | -0.24 (0.155) |
| ***t+10*** | -0.06 (0.052) | 0.01 (0.088) | -0.02 (0.016) | 0.17* (0.071) | 0.46* (0.183) | 0.01 (0.023) | 0.27 (0.257) | -0.14 (0.155) |
| ***t+11*** | -0.1* (0.052) | -0.01 (0.088) | -0.03* (0.016) | 0.23** (0.071) | 0.43* (0.182) | 0.02 (0.023) | 0.46 (0.256) | -0.06 (0.154) |
| ***t+12*** | -0.13* (0.052) | 0.07 (0.087) | -0.06*** (0.016) | 0.35*** (0.07) | 0.43* (0.181) | 0.02 (0.023) | 0.37 (0.254) | -0.15 (0.153) |
| ***t+13*** | -0.06 (0.052) | 0.15 (0.088) | -0.04** (0.016) | 0.27*** (0.071) | 0.51** (0.182) | 0.00 (0.023) | 0.5* (0.255) | -0.01 (0.154) |
| ***t+14*** | -0.11* (0.051) | 0.1 (0.087) | -0.07*** (0.016) | 0.29*** (0.07) | 0.48** (0.181) | -0.01 (0.023) | 0.26 (0.254) | -0.03 (0.153) |
| ***t+15*** | -0.14** (0.052) | 0.09 (0.087) | -0.05** (0.016) | 0.26*** (0.07) | 0.52** (0.181) | -0.01 (0.023) | 0.31 (0.254) | -0.02 (0.153) |
| ***t+16*** | -0.13* (0.052) | 0.02 (0.087) | -0.04** (0.016) | 0.14* (0.07) | 0.56** (0.181) | -0.01 (0.023) | 0.23 (0.254) | -0.03 (0.153) |
| ***t+17*** | -0.15** (0.051) | 0.01 (0.087) | -0.05*** (0.016) | 0.10 (0.07) | 0.55** (0.181) | 0.000(0.023) | 0.28 (0.254) | -0.12 (0.153) |
| ***t+18*** | -0.11* (0.051) | -0.07 (0.087) | -0.04** (0.016) | 0.14* (0.07) | 0.54** (0.181) | 0.01 (0.023) | 0.17 (0.254) | -0.12 (0.153) |
| ***t+19*** | -0.08 (0.052) | 0.02 (0.088) | -0.03 (0.016) | 0.07 (0.071) | 0.50** (0.182) | 0.01 (0.023) | 0.06 (0.256) | -0.32* (0.154) |
| ***t+20*** | -0.07 (0.052) | 0.11 (0.088) | -0.01 (0.016) | 0.13 (0.071) | 0.44* (0.182) | 0.03 (0.023) | 0.05 (0.256) | -0.22 (0.154) |
| ***t+21*** | -0.04 (0.052) | 0.1 (0.088) | -0.01 (0.016) | 0.17* (0.071) | 0.44* (0.182) | 0.01 (0.023) | -0.09 (0.255) | 0.00 (0.154) |
| ***t+22*** | 0.00 (0.052) | 0.07 (0.088) | -0.01 (0.016) | 0.12 (0.071) | 0.45* (0.182) | 0.03 (0.023) | 0.22 (0.255) | -0.19 (0.154) |
| ***t+23*** | 0.00 (0.052) | 0.08 (0.088) | -0.01 (0.016) | 0.18** (0.07) | 0.26 (0.182) | 0.03 (0.023) | 0.18 (0.255) | -0.08 (0.154) |
| ***t+24*** | -0.05 (0.051) | 0.17* (0.087) | -0.01 (0.016) | 0.21** (0.07) | 0.27 (0.181) | 0.03 (0.023) | -0.04 (0.253) | 0.00 (0.153) |
| ***t+25*** | 0.00 (0.051) | 0.08 (0.087) | -0.02 (0.016) | 0.14 (0.07) | 0.28 (0.18) | 0.02 (0.023) | 0.15 (0.252) | 0.05 (0.152) |
| ***t+26*** | -0.03 (0.051) | 0.08 (0.087) | -0.02 (0.016) | 0.18* (0.07) | 0.3 (0.18) | 0.03 (0.023) | 0.01 (0.252) | 0.1 (0.152) |
| ***t+27*** | -0.06 (0.051) | 0.05 (0.086) | -0.02 (0.015) | 0.15* (0.069) | 0.32 (0.178) | 0.03 (0.022) | 0.14 (0.25) | 0.07 (0.151) |
| ***t+28*** | -0.05 (0.051) | 0.09 (0.086) | -0.02 (0.015) | 0.11 (0.069) | 0.37* (0.178) | 0.02 (0.022) | 0.02 (0.25) | -0.1 (0.151) |
| ***t+29*** | -0.08 (0.051) | -0.01 (0.086) | -0.03* (0.015) | 0.16* (0.069) | 0.46* (0.178) | 0.03 (0.022) | 0.09 (0.25) | -0.09 (0.151) |
| ***t+30*** | -0.03 (0.051) | -0.02 (0.085) | -0.02 (0.015) | 0.13 (0.069) | 0.48** (0.178) | 0.02 (0.022) | 0.06 (0.249) | -0.07 (0.15) |
| ***t+31*** | -0.02 (0.05) | 0.05 (0.085) | -0.02 (0.015) | 0.1 (0.069) | 0.46** (0.177) | 0.04 (0.022) | 0.07 (0.249) | -0.03 (0.15) |
| ***t+32*** | -0.02 (0.05) | -0.05 (0.085) | -0.02 (0.015) | 0.08 (0.069) | 0.43** (0.177) | 0.03 (0.022) | -0.01 (0.249) | -0.09 (0.15) |
| ***t+33*** | -0.02 (0.05) | 0 (0.085) | -0.02 (0.015) | 0.14* (0.069) | 0.44** (0.177) | 0.02 (0.022) | 0.11 (0.248) | 0.01 (0.15) |
| ***t+34*** | -0.08 (0.05) | 0 (0.085) | -0.01 (0.015) | 0.17* (0.069) | 0.32 (0.177) | 0.02 (0.022) | 0.07 (0.248) | -0.07 (0.15) |
| ***t+35*** | -0.07 (0.05) | -0.02 (0.085) | -0.01 (0.015) | 0.15* (0.069) | 0.31 (0.177) | 0.03 (0.022) | 0 (0.248) | -0.12 (0.149) |
| **Spring^3^** | 0 | 0 | 0 | 0 | 0 | 0 | 0 | 0 |
| **Summer** | 0.03. (0.018) | -0.03 (0.031) | 0.01 (0.006) | -0.02 (0.025) | 0.11 (0.065) | 0.03** (0.008) | 0.16 (0.091) | -0.01 (0.055) |
| **Fall** | -0.05* (0.019) | -0.01 (0.033) | -0.01 (0.006) | -0.03 (0.026) | 0.29*** (0.066) | -0.02* (0.008) | 0.7*** (0.093) | 0.18** (0.056) |
| **Winter** | -0.12*** (0.016) | 0 (0.028) | -0.03*** (0.005) | 0.07** (0.022) | 0.4*** (0.057) | -0.04*** (0.007) | 0.47*** (0.08) | 0.15** (0.049) |
| **No. of sows** | 0.00* (0.000) | 0.00** (0.000) | 0.00** (0.000) | 0.00 (0.000) | 0.00 (0.000) | 0.00*** (0.000) | 0.00 (0.000) | 0.00 (0.000) |
| **Intercept** | 7.07*** (0.232) | 4.36*** (0.404) | 2.35*** (0.07) | 0.12 (0.316) | 1.14 (0.814) | 3.58*** (0.102) | 0.47 (1.141) | 1.52** (0.689) |
| **Within groups error** | 0.059 | 0.171 | 0.037 | 0.091 | 0.305 | 0.108 | 0.25 | 0.224 |
| **Overall error** | 0.141 | 0.232 | 0.043 | 0.193 | 0.497 | 0.062 | 0.697 | 0.421 |
| **No. Obs.** | 768 | 720 | 768 | 768 | 768 | 768 | 768 | 768 |
| **No. Groups** | 16 | 15 | 16 | 16 | 16 | 16 | 16 | 16 |

Table considers 8 separate log-linear fixed effects regressions (equation (2)) for the number of weaned pigs (WP), number of pre-weaned dead (PWM), number of live births per litter (LS), number of stillbirths per litter (SB), number of sows repeating services (RE), number of sows farrowing (FA), number of sows aborting (AB), and number of sow dead (SM). Numbers indicate estimates, standard errors are reported between parentheses. Asterisk (*), double asterisk (**), and triple asterisk (***) denote 5%, 1%, and 0.1% of significance levels respectively. ^1^ Farm ID #5 was dropped from PWM due to lack of data. ^2^  *t*-12 is used as reference category of *T*. ^3^ Spring is used as reference category of season.
